# The Atopic Dermatitis Patient Journey: From Awareness to Advanced Care

**DOI:** 10.7759/cureus.102538

**Published:** 2026-01-29

**Authors:** Tripti Sharma, Ashwin Balasubramanian, Dhiraj Dhoot, Saiprasad Patil, Hanmant Barkate

**Affiliations:** 1 Department of Global Medical Affairs, Glenmark Pharmaceuticals Limited, Mumbai, IND

**Keywords:** atopic dermatitis, care pathway, expectations, pain points, patient experience, patient journey, quality of life, teledermatology, treatment barriers

## Abstract

Atopic dermatitis (AD) is a chronic, relapsing inflammatory skin disease that significantly impacts the physical, psychological, and social well-being of patients. While clinical guidelines offer structured treatment pathways, real-world patient experiences often differ from clinical expectations due to delayed diagnosis, misinformation, inconsistent care, and emotional distress. This narrative review explores the full spectrum of the AD patient journey, from pre-diagnosis awareness to maintenance therapy and advanced treatment access, highlighting the barriers patients face in navigating care. These include medical, psychological, financial, and systemic obstacles that impair treatment adherence and reduce quality of life (QoL). The importance of early recognition by primary care providers, timely referrals to dermatologists, and patient-centered communication is emphasized. Advancements in topical, systemic, and biologic therapies have transformed the therapeutic landscape; however, fears around steroid use, cost-related limitations, and inadequate care coordination persist. A shift toward proactive management, integration of teledermatology, and patient education programs can bridge the gap between clinical recommendations and real-world outcomes. Understanding the patient journey is essential for developing more inclusive and effective care models that improve long-term disease control and patient QoL.

## Introduction and background

Atopic dermatitis (AD) is a chronic inflammatory skin disorder, also known as atopic eczema, affecting children and adults [1]. It is a heterogeneous disease characterized by recurrent eczematous lesions, intense itching, and discomfort [1,2]. The variable and evolving clinical presentation of AD can contribute to delayed diagnosis or delayed recognition, particularly in nonspecialist settings [3,4]. Onset usually occurs in early childhood and may begin as early as two months of age, although AD can manifest at any point in life [3,5]. Although it is difficult to determine the prevalence of AD, it is estimated to be 5-15% in children and 2-10% in adults, as AD persists into adulthood in almost half of those diagnosed during childhood [1,5]. AD follows a chronic course, often persisting for years, with a relapsing-remitting pattern in many patients [1].

Causes of AD range from genetic to environmental factors and are also associated with an increased risk of comorbidities, such as asthma, allergic rhinitis, and food allergies [5,6]. Environmental elements, such as diet, sleep, allergens, pollution, and humidity, significantly influence the progression of AD, given the skin's constant interaction with its surroundings. Environmental exposures, such as pollution and allergens, can trigger flares and worsen quality of life (QoL) [6]. The complexity and subjectivity of disease severity assessment, along with variability in treatment response, remain key challenges in AD management and contribute substantially to the psychosocial burden experienced by patients and caregivers [7,8].

Patients with AD continue to experience multiple challenges, including persistent itch, variability in treatment response, inadequate disease‑related information, financial constraints, and difficulty navigating care across different healthcare providers, despite significant advances in available therapies [3,9]. Moreover, patients’ health, QoL, sleep, productivity, and interpersonal relationships are negatively impacted. Similarly, children experience an impact on daily activities, school, leisure, and bullying. In this context, AD poses a clear burden on mental and psychological health, with almost half of the patients with severe AD reporting anxiety or moderate-to-severe depression [10,11]. Understanding the patient’s experience and pain points is essential for better adherence to treatments, patient-centered prescribing, higher quality of care, and higher QoL, but studies focused on the patient’s experience are limited, as most studies focus on the clinical and therapeutic process [9,12,13].

Hence, this narrative review constructs a patient‑journey framework by integrating the currently available heterogeneous clinical and experiential evidence in AD. The aim is to provide clinicians with clinically grounded, patient‑centered insights that clarify key decision points, inform practical management considerations, and highlight persistent access and adherence challenges across the continuum of care.

## Review

Methodology

An extensive narrative literature review was conducted using PubMed, Scopus, and Web of Science. Searches combined Medical Subject Headings (MeSH) terms and free-text keywords related to disease characteristics, management, and patient experience, including “atopic dermatitis,” “eczema,” “itch,” “topical therapies,” “systemic therapies,” “emerging treatments,” “proactive management,” “patient journey,” “patient experience,” “quality of life,” “psychological burden,” “unmet needs,” “treatment expectations,” “primary care management,” “specialist referral,” “healthcare barriers,” “digital health,” and “remote care.” Boolean operators (AND/OR) were used to combine terms appropriately.

The search was limited to English-language articles published between January 2000 and March 2025. In line with a narrative review approach, the literature was not restricted to specific study designs and included clinical studies, reviews, guidelines, and qualitative research addressing patient-reported experiences, expectations, barriers to care, and real-world treatment challenges in AD.

Retrieved publications were screened for relevance by the authors and interpreted thematically. Key concepts and recurring clinical and experiential themes were identified and subsequently synthesized within a patient-journey framework. Information was organized into sequential phases reflecting common stages traversed by patients with AD, including pre-diagnosis/awareness, entry into the care circuit, specialist evaluation (dermatologists), initial treatment and maintenance therapy, flare-up management, escalation to systemic and targeted therapies, prevention strategies, and remission/long-term control.

This conceptual structuring approach aligns with a previously published narrative framework describing patient journeys across chronic diseases [14].

Results and discussion

Pre-Diagnosis/Awareness

AD is a condition characterized by “itch,” and because of this, individuals become aware of it due to a persistent itch, redness, or rash. Individuals or parents of children with AD seek information online about their condition, and they encounter misinformation regarding the disease and its management from blogs, promotional websites, and social media and video-sharing platforms. As a result, many initiate self-treatment or over-the-counter (OTC) products that might exacerbate the condition or not help in alleviating their existing symptoms of itching, redness, dryness, burning, and scaling [3]. Awareness campaigns, such as those conducted on World Atopic Eczema Day (September 14), may help improve public recognition of AD and encourage timely care‑seeking for appropriate diagnosis and management. Social media groups, patient support groups, and programs can help in guiding individuals and caretakers alike towards seeking care for AD symptoms [15].

Entry Into the Care Circuit: General Practitioners and Pediatricians

Depending on healthcare system structures, patients may initially consult primary‑level providers in some settings, while in others, where specialist access is readily available, they may directly approach dermatologists for definitive diagnosis and management. Most patients or parents of children suffering from AD would consult the general practitioner (GP) or pediatrician, respectively [4]. Individuals with AD, when the itching, redness, and dry patches become unbearable, hope that the doctor’s visit will yield a straightforward treatment plan that would provide quick relief from symptoms [2]. But as AD is a chronic disease with a complex pathophysiology, it requires specialized care with a long-term plan [12]. GPs, in a bid to control the symptoms, would prescribe moisturizers, antihistamines, and mild steroids, which may work temporarily but do not target the underlying chronic inflammation. A majority of patients with mild AD might benefit from these, but many will only see a partial relief [16].

As symptom duration increases, along with physical symptoms, there is also a decrease in sleep quality and duration. This leads to deterioration of the mental state and QoL of patients with AD. Patients and caregivers feel increasingly lost and exhausted as they continue to seek a solution that works. Patients expect physicians to recognize disease severity and provide a clear management plan. But too often, they leave appointments feeling unheard, confused, or, worse, still symptomatic [9].

Delays in referral to dermatology can result in prolonged symptom burden and ongoing disease activity before specialist assessment. Inefficiencies in early care pathways and variability in disease recognition may contribute to extended periods of trial‑and‑error management prior to appropriate escalation. Strengthening education around AD recognition and referral processes at the primary‑care level may facilitate earlier specialist evaluation and more timely initiation of effective management [4]. Collaborative care models, including teledermatology and structured communication between primary‑level providers and dermatologists, may further help reduce waiting times and improve continuity of care [17]. Inadequate early intervention and delayed access to specialist care can amplify disease burden, with cumulative physical symptoms and a significant psychological impact on patients and caregivers.

Entry Into the Care Circuit: Dermatologists and Specialists

By the time patients present to dermatology, many report limited benefit from prior therapies and a marked decline in QoL. Common complaints include persistent pruritus, inflamed skin, and recurrent flares that disrupt sleep, undermine self‑confidence, and impair daily functioning [18].

Some patients, especially teenagers and young adults, struggle with self-consciousness, stigma, and bullying due to visible patches on their face, arms, or hands [10]. Others deal with chronic discomfort and frequent changes in treatment attempts or medications, hoping for an effective and safe treatment that would provide long-term relief [19].

AD diagnosis is primarily clinical, based on characteristic morphology and distribution, pruritus, age of onset, disease chronicity, and relevant personal/family history of atopy. No single laboratory test is required to establish the diagnosis. Adjunctive biomedical parameters, such as elevated total or allergen‑specific IgE, peripheral eosinophilia, and type‑2 inflammatory markers (e.g., TARC/CCL17), may provide supportive information about disease activity or phenotype, but they are not recommended for routine diagnostic use, and availability varies across health systems. Skin swabs/cultures are reserved for suspected secondary infection during flares, rather than for primary diagnosis. The differential diagnoses include eczematous diseases that can mimic AD, for example, contact dermatitis; conditions with cutaneous hand and foot inflammation, e.g., palmoplantar psoriasis; and immunodeficiency syndromes [1].

After diagnosis, AD can further be staged according to the severity of mild, moderate, or severe. There are various scales and tools that involve objective physician-reported outcomes (e.g., the Eczema Area Severity Index (EASI)) as well as patient-reported outcomes, such as the Itch-Numerical Rating Scale (NRS). This streamlines the identification of appropriate treatment modalities that are most effective for the respective severity of AD [7].

At this stage, patients and caregivers seek more than temporary symptom relief; they expect a clear, sustainable treatment plan that controls flares while maintaining long‑term skin health. Many remain hesitant about prolonged steroid use due to concerns such as skin thinning or dependency [20]. Consequently, there is growing interest in nonsteroidal topical options and targeted therapies that can deliver control with acceptable safety profiles [8]. Dermatology consultations should therefore address these expectations through transparent risk-benefit discussions and individualized planning. Newer biologic therapies, barrier‑repair strategies, and advanced anti‑inflammatory treatments offer hope for patients who have struggled with persistent disease [21,22].

For specialists, it is paramount to educate patients about their condition, reassure them about treatment safety, and provide personalized care plans. AD isn’t just a skin problem; it affects self-esteem, sleep, mental well-being, and overall QoL [11]. They should inform patients about the pathogenic mechanisms, genetic predisposition, barrier dysfunction, and immune dysregulation; predisposing factors for AD; and that they may require more than just symptomatic care. Explaining the Th2-driven inflammation in AD can help patients understand the rationale for advanced treatments, reduce steroid phobia, and improve adherence. A comprehensive approach, combining effective treatments, lifestyle adjustments, and ongoing support, can make a substantial difference in helping patients regain control over their condition and their lives [6].

Initial Treatment and Maintenance Therapy

Following diagnosis, most patients initiate treatment through primary care providers or caregivers.

Non-pharmacological therapy, like daily skin hydration with emollients, is essential to prevent flare-ups. Most guidelines agree that skin hygiene and hydration are the building blocks of initial AD treatment [5,23,24]. However, adherence remains a major challenge due to cost, accessibility, and patient perception [13].

Topical corticosteroids (TCS) are currently recommended as first-line topical treatment for AD due to their overall excellent therapeutic efficacy and the availability of different potency classes and a wide range of formulations [25]. The efficacy of TCS depends on selecting the appropriate vehicle, potency, application frequency, lesion location, treatment duration, and patient preferences.

Low- to mid-potency TCS are recommended when emollients alone are insufficient. However, potent fluorinated corticosteroids are not indicated for use on the face, eyelids, genitalia, intertriginous areas, or in young infants. Topical calcineurin inhibitors (TCIs), such as tacrolimus and pimecrolimus, are alternatives to corticosteroids for AD in sensitive areas such as the face, eyelids, and folds when unresponsive to low-potency topical steroids [26].

Unresponsive Patients and Proactive Management

Patients who do not achieve adequate disease control with regular emollient therapy are typically reassessed by specialists, and treatment is escalated to include topical anti‑inflammatory agents. These may include TCS and TCIs, used either alone or in combination based on disease severity, lesion location, and patient‑specific factors. In recent years, additional nonsteroidal topical agents, such as topical phosphodiesterase‑4 (PDE‑4) inhibitors, have expanded treatment options, particularly for patients requiring long‑term therapy or those with steroid‑sensitive areas.

A proactive treatment strategy is recommended in selected patients to prevent disease flares and maintain long‑term control. This approach involves the intermittent, low‑dose application of TCS, TCI, or other non‑steroidal topical agents to previously affected skin areas, while continuing regular use of emollients on unaffected skin. Proactive therapy is typically continued for a defined period following lesion clearance to reduce relapse risk. For patients with persistent moderate‑to‑severe disease despite optimized topical therapy, escalation to systemic non‑biologic agents or targeted biologic therapies may be necessary. This stepwise approach enables patients to move from reactive flare management toward sustained disease control and active self‑management of AD [27,28].

Escalation to Systemic and Targeted Therapies (Experts Care)

Patients with moderate-to-severe AD often require escalation to topical and systemic therapies. Systemic immunosuppressants such as cyclosporine, methotrexate, and azathioprine are used in moderate-to-severe AD but require close monitoring due to potential adverse effects.

Targeted therapies: Dupilumab (IL-4/IL-13 inhibitor) and Janus kinase (JAK) inhibitors have transformed AD treatment, reducing symptoms like itching and inflammation. They offer rapid symptom relief but require further long-term safety evaluation. For those with moderate-to-severe disease who are refractory, intolerant, or unable to use mild-to-high-potency topical treatments, dupilumab can be given to patients >=6 months of age, and selective JAK1/2 inhibitors like abrocitinib can be given to those >=12 years of age [29,30].

Patients face barriers to oral therapies, including cost, drug side effects, immunosuppression-related side effects, and lifestyle disruptions. The AD therapeutic landscape is evolving, with biologics offering durable control in moderate-to-severe cases. However, long-term effectiveness, safety, and accessibility remain key considerations.

Barriers to Treatment

Despite advances in treatment, patients face a wide array of barriers. These challenges are categorized below to guide system-level improvements based on the Eczema Society of Canada's Atopic Dermatitis Patient Journey Report (2020) in Table 1 [26].

**Table 1:** Challenges and barriers to treatment in atopic dermatitis

Category	Key Barriers
1. Medical Barriers	Perceived lack of treatment effectiveness, burning, stinging, or irritation from topicals, steroid phobia, delayed diagnosis or mismanagement, or early treatment discontinuation.
2. Psychological & Emotional Barriers	Low trust in healthcare providers (only 43% trust treatment recommendations), emotional exhaustion, and treatment fatigue. Depression, anxiety, low self-esteem, and frustration during flares (reported by 80%).
3. Financial & Accessibility Barriers	Prohibitive cost of biologics and systemic therapies, limited availability of phototherapy, insurance and reimbursement issues, inadequate access to specialists in remote areas, and long wait times for referral.
4. Systemic & Healthcare Barriers	Poor coordination among healthcare providers, reactive care (treatment changes only during flares), inconvenient or delayed appointments, lack of standardized treatment protocols, and insufficient patient education.
5. Alternative Treatment & Misinformation	Thirty-five percent of patients seek alternative therapies due to distrust. Seven percent abandon prescribed treatment for unproven remedies. Conflicting and misleading online information increases treatment hesitancy.

Flare-Up Management

Patients with moderate to severe AD experience an average of nine flare episodes a year, with flare intensity and duration (days to weeks) typically increasing with underlying AD severity [31,32]. The most common symptom of atopic flare‑ups is itching, which can be severe [2,6]. Other common symptoms include red, dry patches of skin [2]. Additional features during flare‑ups may include erythematous, dry skin lesions that can ooze or weep clear fluid and may bleed when excoriated [2,19]. Clinical management of AD should account for disease severity and distribution, underlying pathogenic mechanisms, and individual patient factors such as age, comorbidities, and trigger profiles, with a primary goal of preventing disease flares [1,5,23]. For the measurement of clinician‑reported disease severity, the Scoring of Atopic Dermatitis (SCORAD) and the EASI are among the most widely used tools [5,7,19]. Compared with the EASI, which quantifies the extent and severity of skin involvement, the SCORAD incorporates both objective parameters (such as lesion extent and intensity) and subjective patient‑reported symptoms, including itch and sleep disturbance [19,33]. Regardless of disease severity, however, appropriate skincare must be adhered to, with the use of emollients and mild skin cleansers providing the basis for continued skincare and addressing the dysfunctional epidermal barrier with hydrating/lubricating topical treatment, along with patient education programs and the avoidance of trigger factors [1,8,9,22,23].

For active, visible skin lesions in AD, topical anti‑inflammatory therapy involves the use of TCS, with TCIs as alternatives [5,23,25,26]. Topical steroids are the first‑line anti‑inflammatory treatment for acute flares and should be applied at the first sign of an acute flare and should not be spared [20,25]. If topical treatments have failed or are inadequate to control AD flares, other therapeutic options should be considered [5,23,24]. The choice of systemic immunosuppressive treatment for flare management should be based primarily on the rapid onset of action [24]. Systemic antihistamines may be used in acute flares, but evidence supporting their use in targeting itch is very weak, and their effects on AD‑related itch and lesions are limited [34]. Topical antiseptics (such as potassium permanganate soaks or dilute bleach baths), as well as topical antibiotics (e.g., mupirocin or fusidic acid) and systemic antibiotics when indicated, are recommended when acute flares are complicated by clinical signs of bacterial infection, including oozing, crusting, or pustules [23,32,35]. Secondary infections of lesions caused by fungal or viral agents (eczema herpeticum) should also be investigated early and treated promptly if present [36]. After acute flares are controlled, preventing recurrence becomes the primary objective [23,27,28].

Prevention Strategies

Preventive strategies for AD are largely non-pharmacological measures that aim to reduce flares and maintain remission or prevent the increase in disease severity [23,36]. The first measure is preserving the skin barrier, which can be achieved through early and consistent use of emollients (e.g., ceramides, liquid paraffin) to keep the skin hydrated and resilient [22,23]. This can help prevent dryness and micro-injury that may trigger inflammation [1,23]. Avoiding triggers such as harsh cleansers, allergens, and environmental irritants is equally important, as these factors can damage the skin barrier and precipitate flare-ups [1,2,23]. Addressing immunological dysregulation may involve supporting a balanced immune response through the skin-gut axis, including attention to nutrition and products containing pre-, pro-, and post-biotics, which may influence immune pathways [37,38]. Finally, minimizing bacterial colonization, particularly by *Staphylococcus aureus*, is important as it can exacerbate inflammation; strategies include maintaining good hygiene and, in selected cases, adjunctive antiseptic measures such as potassium permanganate (KMNO₄) and dilute bleach baths [32,35,39]. While these interventions are promising, their effectiveness varies between individuals and across studies, highlighting the need for personalized prevention plans [36]. Specialists can emphasize to patients and caregivers that preventive measures are not only intended for symptom control but may also help reduce the risk of progression along the atopic march, from AD in infancy to food allergy, allergic rhinitis, and asthma [40]. Educating families about early barrier repair, proactive therapy, and microbiome modulation can empower them to adopt strategies that may interrupt this trajectory [6].

Remission Phase Management

Moisturizers will always be the cornerstone of AD in the remission phase and should be continued to prolong the remission period and spare topical steroid use. For patients and families with poor QoL, sleep disturbance, avoidance of social activities, worsening anxiety and depression, psychological counselling, patient support, and educational programs can be implemented [41].

**Figure 1:**
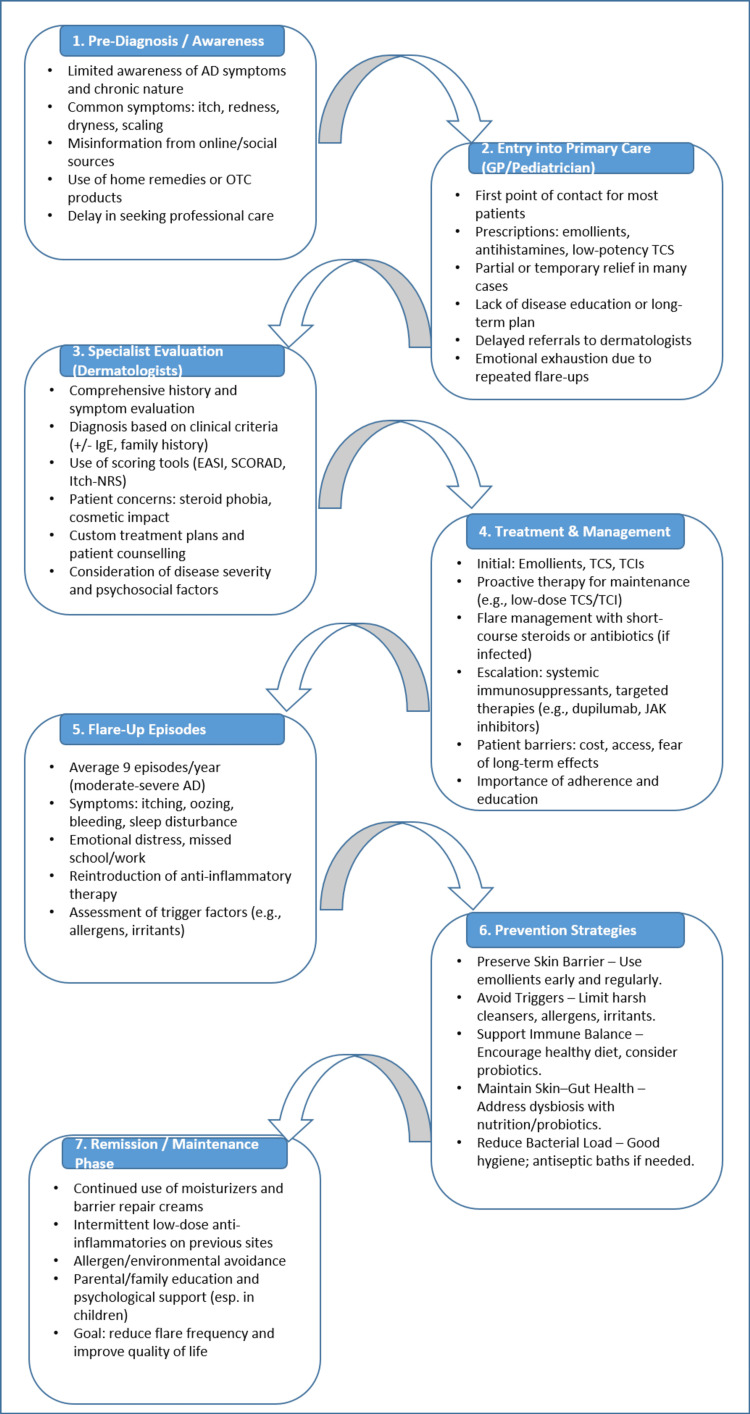
A patient’s journey with atopic dermatitis AD: atopic dermatitis; OTC: over-the-counter; TCS: topical corticosteroids; GP: general practitioner; EASI: Eczema Area Severity Index; SCORAD: Scoring of Atopic Dermatitis; NRS: Numerical Rating Scale; TCIs: topical calcineurin inhibitors; JAK: Janus kinase

The AD patient journey has been summarized in Figure 1. This figure gives an overview and traces the clinical trajectory of AD, from initial diagnosis through to achieving sustained remission.

## Conclusions

AD is a chronic, multifaceted skin disorder that significantly affects patients' QoL. The patient journey in AD is marked by numerous challenges, beginning with delayed diagnosis and misinformation, progressing through inadequate primary care management, and often culminating in frustration due to ineffective treatments or barriers to accessing advanced therapies. The physical, emotional, and psychological burden of AD is profound, influencing sleep, mental well-being, social interactions, and daily functioning.

The importance of early intervention, patient education, and a multidisciplinary approach cannot be overstated. GPs and pediatricians play a crucial role in recognizing AD symptoms early and guiding patients toward dermatological expertise. Dermatologists, in turn, must provide individualized, evidence-based treatment strategies that incorporate not only pharmacological therapies but also proactive and non-pharmacological approaches, emphasizing skin barrier repair and long-term disease control.

Advancements in systemic and biologic therapies have revolutionized AD management, offering hope to patients with moderate-to-severe disease. However, access to these treatments remains a significant challenge due to high treatment costs, limited insurance coverage or reimbursement, patient concerns regarding potential adverse effects, and variability in the availability of specialist care and healthcare infrastructure across settings. Addressing steroid phobia, improving adherence to treatment regimens, and ensuring patient-centric care models can further enhance therapeutic outcomes.

A comprehensive and patient-centered approach, one that integrates medical, psychological, and social support, is essential for improving the overall patient experience. By identifying barriers within the patient journey and implementing strategies to overcome them, we can work toward optimizing AD management and ultimately enhancing patients' QoL. Future research should focus on bridging gaps in care, expanding access to novel therapies, and fostering a more inclusive healthcare model that prioritizes both disease control and holistic well-being. They must also integrate patient voices into guideline development and expand access to emerging therapies.
